# Differences in Platelet Indices between Healthy Han Population and Tibetans in China

**DOI:** 10.1371/journal.pone.0067203

**Published:** 2013-06-24

**Authors:** Qian Niu, Ruke Zhang, Min Zhao, Sugen Zeng, Xunbei Huang, Hong Jiang, Youfang An, Luwen Zhang

**Affiliations:** 1 Department of Laboratory Medicine, West China Hospital, Sichuan University, Chengdu, The People’s Republic of China; 2 Department of Laboratory Medicine, People’s Hospital of The Tibet autonomous region, Lhasa, The People’s Republic of China; 3 Department of Laboratory Medicine, Hospital of the Office of The Tibet autonomous region in Chengdu, Chengdu, The People’s Republic of China; Robert Wood Johnson Medical School, United States of America

## Abstract

**Introduction:**

The present data on the evaluation of platelet (PLT) parameters in Chinese Han population and Tibetans are still limited. The objective of this study was to determine the differences in common PLT indices between Han population and Tibetans in China, through a large-scale investigation of healthy people.

**Methods:**

2131 Han people from Chengdu Plain, 1099 Tibetans from Qinghai-Tibet Plateau and 956 Plateau Han migrants were included in this study. All the subjects were healthy people through the health screening. PLT indices were measured with Sysmex XE-2100 and XT-1800i blood cell automatic analyzer.

**Results:**

Compared with Han people in Chendu Plain, Tibetans had higher PLT count (*P*<0.01) but lower mean platelet volume (MPV), platelet distribution width (PDW) and platelet-large cell ratio (P-LCR) (*P*<0.01); while Plateau Han migrants had lower PLT count, MPV and P-LCR (*P*<0.05). When compared with Tibetans, Plateau Han migrants had lower levels of mean PLT count but higher PDW and P-LCR (*P*<0.05).

**Conclusions:**

There are ethnic differences in PLT indices between Chinese Han population and Tibetans. Based on this finding, it would be reasonable to conduct formal prospective studies to determine the clinical significance of these differences and to explore the effects of genetic background on these indices.

## Introduction

Chinese Tibetans have lived in a very high altitude (mean 4500 m) and hypoxic environment at the Qinghai-Tibet Plateau for thousands of years. They thus have a suite of distinctive physiological traits that enable them to tolerate the extreme environment, such as decreased arterial oxygen content [1] and limited increased red blood cell (RBC) counts and hemoglobin concentration [2]. However, there are still some other physiological changes in Tibetans, which have not been reported.

Platelets (PLTs) are well known to play an important role in hemostasis and blood coagulation. Once defects happen in amount and/or function of PLTs, the risk of bleeding will increase [3]. Clinical monitoring of PLT amount and activity mainly rely on the determination of PLT indices including PLT count, Mean platelet volume (MPV), platelet distribution width (PDW) and platelet-large cell ratio (P-LCR), among which PLT count is the most common and important parameter. However, MPV, PDW and P-LCR always tend to be neglected in the clinical application. In fact, these indices, especially MPV and PDW, are reported to correlate with platelet function and may be more sensitive indices than platelet count as markers of clinical interest in various disorders, including some cardio- and cerobro-vascular diseases [4∼6].

Chronic hypoxia is demonstrated as a risk factor of multiple cardio- and cerobro-vascular disorders, such as congestive heart failure [7] and stroke [8]. Meanwhile, high altitude has been observed to a hypercoagulable state, thus predisposing to thromboembolic events [9]. However, it’s interesting that there are no evidences showing higher prevalence of vascular disease in Chinese Tibetans than people living at low altitude. Even, stroke incidence rate was lower in Tibetans in Lhasa than Han people there and in other parts of China [10]. All these suggest that some certain physiological factors may be involved in the un-increased incidences of those vascular diseases. In view of the important role of PLT in cardiovascular and cerebrovascular diseases, we want to determine if there are differences in PLT indices between Chinese Tibetans and Han population.

## Materials and Methods

This study was approved by the Ethics Committee of Chinese Human Genome and the Ethics Committee of West China Hospital, and written informed consent was obtained from all participants.

### Participants

2131 Han people from Chengdu Plain, 1099 Tibetans from Qinghai-Tibet Plateau and 956 Plateau Han migrants were included in this study. All volunteers have lived in their residence for more than 3 years. Every subject underwent an interview with a physician using a life-health questionnaire and a physical examination form.

Exclusion criteria included body mass index (BMI) ≥28, high blood pressure (systolic number≥140 mmHg and/or diastolic number≥90 mmHg), alcohol assumption>30/g, tobacco use>20 cigarettes, drugs intake within 2 weeks, surgery within 6 months, blood donor or transfusion within 4 months, pregnancy, less than 1 year after childbirth, hypothyroidism and hyperthyroidism, diabetes, atherosclerosis and vascular disease, cardiopathy, chronic nephropathy, hepatobiliary disease, allergic diseases, hematological disease, myopathy, autoimmune disease, burns and muscle trauma, the presence of acute and chronic infection, plasma fasting glucose>7.0 mmol/L, serum creatinine>120 µmol/L, serum creatine kinase>400 U/L, positive hepatitis B surface antigen, positive anti-hepatitis C virus antibody, positive anti- immunodeficiency virus antibody and positive urinalysis.

After strict screening, a total of 4186 blood samples were obtained. Samples were collected in West China Hospital of Sichuan University and Hospital of Chengdu Office, People's autonomous Government of Tibetan from October 2011 to May 2012. Demographic features of all participants were provided in Table 1.

**Table 1:** Demographics of three group subjects.

Group	*n*	Male/female	Age (years)	*P* value
Han people in plain	2131	856/1275	40.1±12.4	>0.05
Tibetans in Plateau	1099	508/591	45.3±13.1	>0.05
Plateau Han migrants	956	430/526	46.4±13.9	>0.05

Values are expressed as mean±SD or number.

### Methods

#### Blood samples

For all participants, a 2-ml fasting blood sample was drawn into a BD Vacutainer tube with EDTA-K_2_ anticoagulant at 8∶00–9∶00 a.m. Complete blood Counts (CBC) were all completed within 30 minutes after blood drawing.

#### Instruments

XE-2100 blood cell automatic analyzer in West China Hospital and XT-1800i blood cell automatic analyzer in hospital of Chengdu Office, People's autonomous Government of Tibetan were all purchased from Sysmex, Japan.

#### Standardization and assurance of quality control

Before and after samples testing, three levels of controls (e-CHECK for XE and e-CHECK for XT were purchased from Sysmex, Japan) were performed. Clinical laboratories of two hospitals regularly participated in proficiency test (PT) of Chinese Ministry of Health, and laboratory of West China Hospital has gained accreditation from the College of American Pathologist.

### Statistical Analysis

Data were analyzed using SPSS 17.0 software (Chicago, IL). *ANOVA* and *Student-Newman-Keuls* were performed to detect the difference among groups. Spearman's correlation was used as a test of correlation between two continuous variables. *P* Values less than 0.05 were considered significant. 95% confidence interval (CI) of each parameter was expressed by calculating the 2.5^th^–97.5^th^ percentile.

## Results

### Basic Characteristics of All Subjects

There were no significant differences in either age or gender among the three groups of subjects (Table 1).

### Analysis of PLT Indices

**Figure 1:**
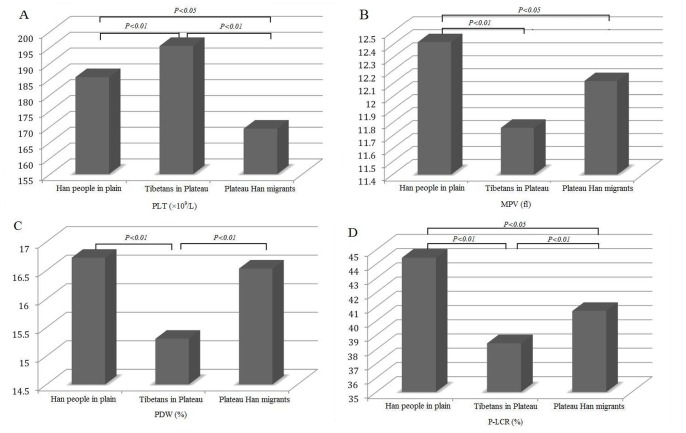
Analysis of PLT indices in three groups. A: Analysis of the mean PLT count; B: Analysis of MPV; C: Analysis of PDW; D: Analysis of P-LCR.

As shown in Fig. 1A∼D, among three groups, Tibetans in Plateau had the highest mean PLT count and the lowest MPV, PDW and P-LCR (*P*<0.01). When compared with Plateau Han migrants, mean PLT count, MPV and P-LCR of Han people in plain was significantly higher (*P*<0.05), while there was no obvious difference of PDW between these two groups (*P*>0.05). Specific data and 95% CIs see Table 2.

**Table 2:** Analysis of PLT indices in three groups.

Group	PLT (×10^9^/L)	MPV (fl)	PDW (%)	P-LCR (%)
Han people in plain	185.59±52.20* (83∼268)	12.42±1.20* # (10.1∼22.5)	16.72±3.17* (10.5∼27.2)	44.44±9.36* # (26.1∼70.5)
Tibetans in Plateau	195.42±57.63# (82∼278)	11.76±1.11 (9.6∼21.3)	15.30±2.79 (9.8∼25.1)	38.42±8.01# (22.7∼61.1)
Plateau Han migrants	169.32±54.52 (62∼232)	12.12±1.08 (10.0∼22.1)	16.53±3.02* (10.6∼27.1)	40.69±7.57 (25.9∼66.5)

Data are expressed as mean±SD with 95% CI.

* *P*<0.01 *vs* Tibetans in Plateau;

# *P*<0.05 *vs* Plateau Han migrants.

### Correlation Analysis

**Figure 2:**
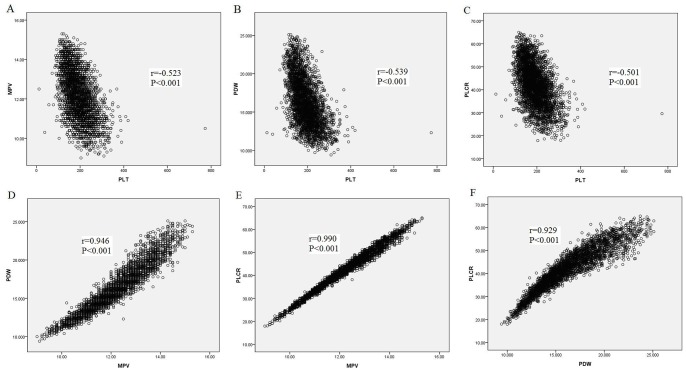
Spearman's correlation analysis. A: The PLT count was negatively correlated with MPV (r = −0.523, *P*<0.001); B: The PLT count was negatively correlated with PDW (r = −0.539, *P*<0.001); C: The PLT count was negatively correlated with P-LCR (r = −0.501, *P*<0.001); D: MPV was positively correlated with PDW (r = 0.946, *P*<0.001); E: MPV was positively correlated with P-LCR (r = 0.990, *P*<0.001); F: PDW was positively correlated with P-LCR (r = 0.929, *P*<0.001).

As shown in Fig. 2, the PLT count was negatively correlated with MPV (r = −0.523, *P*<0.001, Fig. 2A), and PDW (r = −0.539, *P*<0.001, Fig. 2B) as well as P-LCR (r = −0.501, *P*<0.001, Fig. 2C). While MPV were positively correlated with either PDW (r = 0.946, *P*<0.001, Fig. 2D) or P-LCR (r = 0.990, *P*<0.001, Fig. 2E). As well, PDW was positively correlated with P-LCR (r = 0.929, *P*<0.001, Fig. 2F).

## Discussion

Although a various of studies allowed researchers to realize the distinctive physiological traits of Tibetans in China, the physiological changes of PLT indices in Tibetans are still unknown. Our results demonstrated that there were ethnic differences in PLT indices between healthy Chinese Tibetans and Han population. Tibetans in Plateau had higher mean PLT count but lower MPV, PDW and P-LCR as compared with either Han population in Chengdu Plain or Plateau Han migrants.

Several studies have independently demonstrated that high-altitude hypoxia exposure had great impact on the generation or function of not only red blood cells (RBCs) but also platelets in the blood [11]. According to the reports, although short-term hypoxia exposure increased levels of a number of haematological parameters including PLT number [12], long-term hypoxia and high-altitude exposure could obviously decrease the PLT count, due to the enhancement of the activation and consumption of PLTs [11]. Relatively lower PLT concentration can reduce blood viscosity to a certain extent and thus is good for microcirculation perfusion [13]. This is important for Tibetans to adapt to the extreme hypoxia environment at high-altitude. Our results verified that the mean PLT count of Han people moved to the Plateau decreased significantly when compared to Han population living in the Plain, suggesting again that high-altitude exposure could reduce the PLT number.

However, it was interesting to find that Tibetans living in Plateau had a higher mean PLT count than that of Han people in Plain, which was not be consistent with the previous studies. Then we compared the 95% confidence interval (CI) of PLT count in this study with the reference range of PLT count in Chinese healthy adults,and results indicated that the mean PLT number of Han people living in Chengdu Plain [(83∼268)×10^9^/L] was lower than that of national average level [(125∼325)×10^9^/L] (Data was released by Chinese Ministry of Health). Decrease of mean PLT number of Han people in Plain made the PLT count of Tibetans seems to be higher. But, the reasons why Han people in Chengdu Plain had lower PLT number are still unclear, and exploring the specific mechanisms on the basis of environments and genetics is our next work.

MPV is a simple indicator of platelet size and has been known to be a marker of platelet activation. According to recent studies, MPV is considered a link between inflammation and thrombosis in multiple cardiovascular and cerebrovascular disorders including stroke, peripheral artery disease, and coronary heart disease [14∼16]. In our study, MPV of Tibetans was obviously lower than that of both Han people in Plain and Plateau Han migrants, which could be the reason why Tibetans did not live with high prevalence of vascular disease.

PDW is an index reflecting the heterogeneity of platelets, while P-LCR is the proportion of large platelets. Generally, the more large platelets exist in blood, the higher MPV and PDW are [17]. Our results indicated that Chinese Tibetans had lower P-LCR and PDW, which were in accord with the change of MPV. The correlation analysis verified that MPV and PDW, MPV and P-LCR, PDW and P-LCR were positively correlated, respectively. While, these three parameters were all negatively correlated with PLT count, suggesting the reciprocal relationship between PLT count and other PLT indices.

There were also differences in the PLT indices between Han people in Plain and Plateau Han migrants in this study, although these two group subjects had the same genetic background. Results suggested the remarkable impact of environment on PLT indices.

### Conclusions

To our knowledge, this is the first report to explore the ethnic differences in PLT indices between healthy Chinese Tibetans and Han population in so large-scale. The model of PLT indices in Tibetans was higher PLT count accompanied by lower MPV, PDW, and P-LCR, which was possibly the result of their adaption to the extreme environment in Qinghai-Tibet Plateau. The PLT count of Han people living in Chengdu Plain was lower than Chinese mean level, and this phenomenon was worth further research. Genetic and environment were two main factors causing physiological variations, therefore,unique reference ranges of some routine inspection items should be established for people of different ethnic groups or in different regions.
